# Effects of Drought Stress Induced by Hypertonic Polyethylene Glycol (PEG-6000) on *Passiflora edulis* Sims Physiological Properties

**DOI:** 10.3390/plants12122296

**Published:** 2023-06-12

**Authors:** Ying Qi, Lingling Ma, Muhammad Imran Ghani, Qiang Peng, Ruidong Fan, Xiaojing Hu, Xiaoyulong Chen

**Affiliations:** 1 College of Agriculture, Guizhou University, Guiyang 550025, China; 2 International Jointed Institute of Plant Microbial Ecology and Resource Management in Guizhou University, Ministry of Agriculture, China Association of Agricultural Science Societies, Guiyang 550025, China; 3 Guizhou-Europe Environmental Biotechnology and Agricultural Informatics Oversea Innovation Center, Guizhou Provincial Science and Technology Department, Guizhou University, Guiyang 550025, China; 4 College of Life Sciences, Guizhou University, Guiyang 550025, China

**Keywords:** *Passiflora edulis* Sims, drought stress, hypertonic polyethylene glycol, physiological characteristics

## Abstract

Passion fruit is known to be sensitive to drought, and in order to study the physiological and biochemical changes that occur in passion fruit seedlings under drought stress, a hypertonic polyethylene glycol (PEG) solution (5%, 10%, 15%, and 20%) was used to simulate drought stress in passion fruit seedlings. We explored the physiological changes in passion fruit seedlings under drought stress induced by PEG to elucidate their response to drought stress and provide a theoretical basis for drought-resistant cultivation of passion fruit seedlings. The results show that drought stress induced by PEG had a significant effect on the growth and physiological indices of passion fruit. Drought stress significantly decreased fresh weight, chlorophyll content, and root vitality. Conversely, the contents of soluble protein (SP), proline (Pro), and malondialdehyde (MDA) increased gradually with the increasing PEG concentration and prolonged stress duration. After nine days, the SP, Pro and MDA contents were higher in passion fruit leaves and roots under 20% PEG treatments compared with the control. Additionally, with the increase in drought time, the activities of antioxidant enzymes such as peroxidase (POD), superoxide dismutase (SOD) and catalase (CAT) showed an increasing trend and then a decreasing trend, and they reached the highest value at the sixth day of drought stress. After rehydration, SP, Pro and MDA contents in the leaves and roots of passion fruit seedlings was reduced. Among all the stress treatments, 20% PEG had the most significant effect on passion fruit seedlings. Therefore, our study demonstrated sensitive concentrations of PEG to simulate drought stress on passion fruit and revealed the physiological adaptability of passion fruit to drought stress.

## 1. Introduction

In recent decades, rainfall patterns have changed, owing to the impacts of global warming, and droughts have become more frequent in many agricultural areas [1]. The increased frequency of droughts can have significant adverse effects on plant growth and development. Therefore, it is important to characterize the effects of drought stress on higher plants, which is a key factor in assessing the future suitability of changing environments for growing higher plants [2]. Moreover, a growing population and intensified human activities lead to higher water consumption. Therefore, water shortage has become one of the world’s major environmental problems that limit crop productivity [3]. It is estimated that almost half of all land in China is arid or semiarid. Drought is a major issue expecially in the northwest and southwest regions of China. Even though the southern region of China receives ample rainfall, this region still faces seasonal drought, which is one of the key factors hindering plant growth and development in this area [4]. Water scarcity causes changes in the morphological, physiological, and biochemical metabolic pathways of plants [5]. 

Drought stress is detrimental to germination and seedling growth. It diminishes photosynthetic pigments, membrane functions, and enzyme activity, thus significantly reducing crop yield. The vegetative phase and the beginning of flowering are two sensitive stages of development [6,7]. Plants can naturally employ a complex network of different mechanisms at different growth stages of development to attenuate the effects of drought stress [5]. Drought stress causes the overproduction of reactive oxygen species (ROS) in the plant, which in turn causes oxidative damage and a shift in the activity of crucial metabolic pathways. Increasing accumulation of ROS in the plant is associated with impaired membrane stability and cell functions [8,9]. To prevent metabolic damage from excess ROS, plants activate their antioxidant systems, which are found throughout the cell and are responsible for cell stabilization by eliminating excess ROS [6,7,9].

Passion fruit (*Passiflora edulis* Sims) is an herbaceous vine of the Passifloraceae family and the genus *Passiflora*, native to southern Brazil [10,11]. Passion fruit is rich in minerals and various vitamins [12], and essential fatty acids such as linoleic, oleic, and palmitic acids are found in its seeds [13]. Some passion fruit species are colorful and can be used as ornamental plants [14]. Passion fruit has culinary, medicinal, and ornamental value, with great commercial potential [15,16]. However, the growth, yield, and quality of passion fruit are limited by a variety of environmental factors [17,18]. As shallow-rooted plants, passion fruits are sensitive to soil moisture levels and are affected by water logging and drought. Insufficient water supply leads to malnutrition and reduced flowering of passion fruit [19]. Drought is an important factor that affects plant growth and development [20]. Water deficiency can cause physiological and biochemical changes in passion fruit, such as leaf curling and yellowing [21]. Passion fruit is typically cultivated in the open field and is widely distributed throughout the country in regions such as Guangxi, Yunnan, Fujian, and Guizhou, with a wide planting area. However, climate change and frequent natural disasters, including drought and flooding, have resulted in severe loss of passion fruit production. Due to these factors, the productivity of passion fruit in these regions was significantly influenced [22]. For instance, drought affects key growing regions to varying degrees in early spring, resulting in poor growth and crop damage or even crop death in some areas, ultimately reducing passion fruit yield. The effects of drought have severely limited the sustainable development of the passion fruit industry.

Polyethylene glycol (PEG-6000) refers to a class of polymers with a defined molecular weight that is nontoxic, nonionic, and induces drought stress without causing direct cellular or physiological injury. PEG can alter the osmotic potential of nutrient medium in a relatively regulated manner [23]. Moreover, it has strong water absorption properties and can dehydrate plant cells to simulate drought stress [24]. Several studies have been conducted on the detrimental effects of drought stress on seed germination, seedling, and root growth in various crops, including sorghum and wheat [25,26]. However, little information is known about the effects of drought stress on passion fruit seedlings, particularly in relation to PEG-induced drought stress. We postulated that under drought stress, different PEG levels have different effects on physiological and biochemical parameters and that these effects become more pronounced with increasing concentration and duration. However, some recovery may occur after rehydration, and the recovery ability of plants at different PEG levels is different. Therefore, the aim of this study was to investigate the physiological changes in chlorophyll, lipid peroxidation, resilient antioxidant enzymes, root vigor, and osmotic regulatory substances in passion fruit seedlings under PEG-mediated drought stress and to investigate the physiological adaptations of passion fruit seedlings under drought stress.

## 2. Results

### 2.1. The Effects of Drought Stress Induced by Different Concentrations of Polyethylene Glycol (PEG) on the Fresh Weight of Passion Fruit Seedlings

The growth parameters of passion fruit seedlings subjected to various levels and durations of drought stress were studied. Different concentrations of PEG had a significant impact on the growth of passion fruit seedlings (Table 1). Fresh biomass increased continuously with treatment time, reaching its highest weight on day 9. The fresh biomass of the control (CK) was the highest, followed in descending order by that of 5% PEG-6000, 10% PEG-6000, 15% PEG-6000, and 20% PEG-6000 treatments. Compared with CK, 15% PEG-6000 and 20% PEG-6000 treatments had significantly lower measurements, with 18.73% and 25.27% fresh weight above the ground, respectively, and 37.71% and 41.71% fresh weight below the ground, respectively. 

### 2.2. The Effects of Drought Stress Induced by Different Concentrations of Polyethylene Glycol (PEG) on Physiological Properties of Passion Fruit Seedlings

#### 2.2.1. Changes in Chlorophyll Content

**Figure 1:**
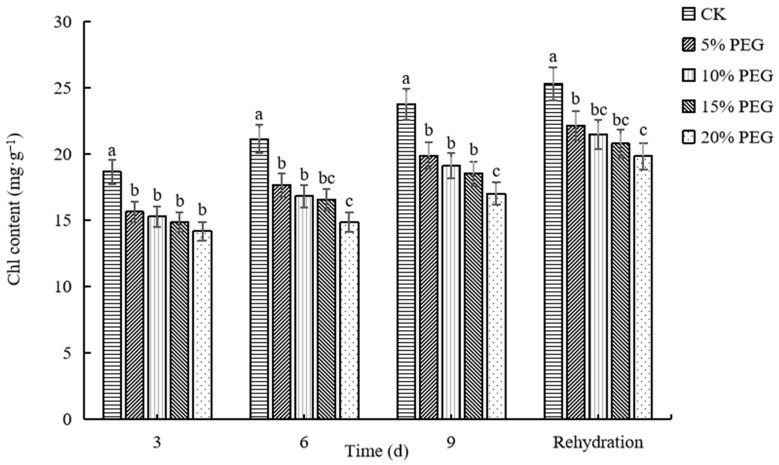
The effects of drought stress induced by different concentrations of polyethylene glycol (PEG) on the Chl content of passion fruit seedlings. Different lowercase letters in the same column indicate significant differences among treatments at the 0.05 level.

The chlorophyll (Chl) content of each treatment showed an increasing trend with time, and the higher the PEG concentration, the smaller the increase in Chl content (Figure 1). The Chl content of each treatment was significantly lower than that of CK. After 9 days of drought, Chl content was the highest in CK, followed in descending order by that of 5% PEG-6000, 10% PEG-6000, 15% PEG-6000, and 20% PEG-6000 treatments, with the lowest Chl content at only 16.98 mg·g^−1^ in 20% PEG-6000 treatment. The Chl content of 20% PEG-6000 treatment after rehydration was 39.67% lower than that of CK. These findings suggest that the ability of passion fruit seedlings to synthesize photosynthetic pigments was poor under drought stress conditions.

#### 2.2.2. Changes in Root Vitality 

**Figure 2:**
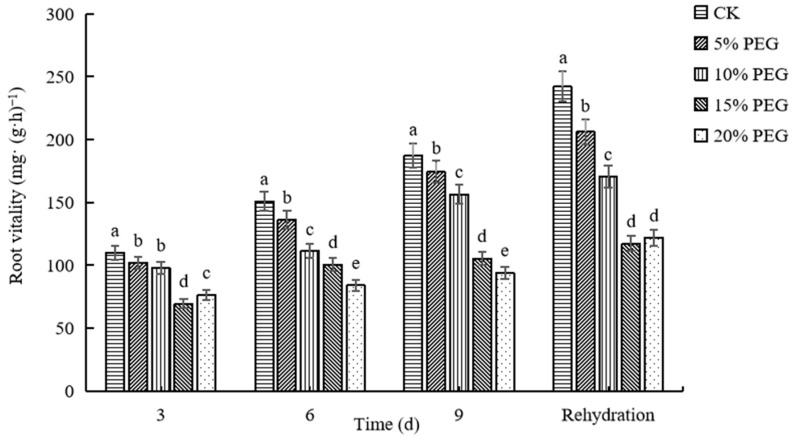
The effects of drought stress induced by different concentrations of polyethylene glycol (PEG) on root vitality of passion fruit seedlings. Different lowercase letters in the same column indicate significant differences among treatments at the 0.05 level.

Root vitality under different concentrations of PEG increased with the increase in time, but under the same treatment time, root vitality decreased with the increase in concentration (Figure 2). The root vitality of CK was the highest, followed in descending order by that of 5% PEG-6000, 10% PEG-6000, 15% PEG-6000, and 20% PEG-6000 treatments. On the 9th day of drought, the root vitality of 20% PEG-6000 treatment was 106.60% lower than that of CK. These results indicate that passion fruit seedlings under drought conditions can increase the water absorption area and enhance their ability to absorb soil nutrients by increasing their number of lateral roots, thereby enhancing their root vitality and adapting to the external arid environment. However, with the increase in PEG concentration, the root viability increased slowly, indicating that PEG inhibited the growth of passion fruit seedlings to a certain extent.

#### 2.2.3. Changes in Soluble Protein and Proline Contents 

**Figure 3:**
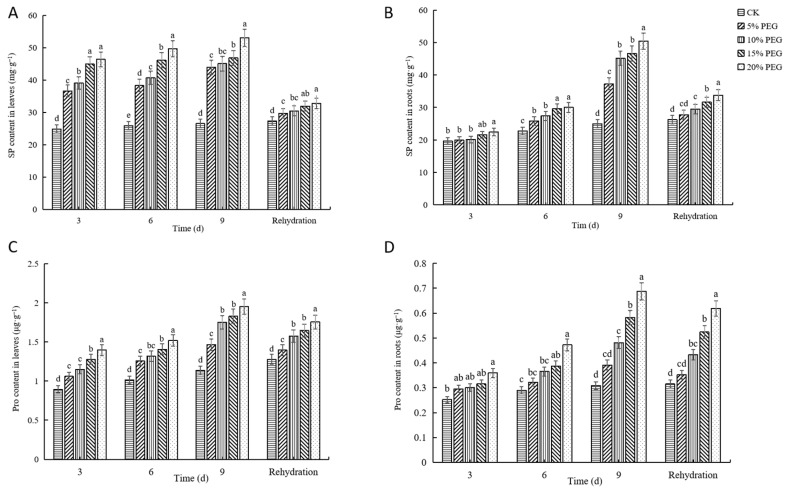
The effects of drought stress induced by different concentrations of polyethylene glycol (PEG) on soluble protein (SP) and proline (Pro) content in passion fruit seedlings: (**A**) SP content in leaves. (**B**) SP content in roots. (**C**) Pro content in leaves. (**D**) Pro content in roots. Different lowercase letters in the same column indicate significant differences among treatments at the 0.05 level.

The soluble protein (SP) and proline (Pro) content in passion fruit seedling leaves and roots in each treatment increased with time and reached the highest value on day 9 (Figure 3). The SP and Pro content of 20% PEG-6000 treatment was the highest, followed in descending order by that of 15% PEG-6000, 10% PEG-6000, 5% PEG-6000 treatments, and CK, and the values for each treatment were significantly different from those of CK. Furthermore, the SP content of 20% PEG-6000 treatment increased by 100.04% and 101.96% in leaves and roots, respectively, compared with that of CK, and the Pro content increased by 71.69% and 122.76% in leaves and roots, respectively, compared with that of CK.

#### 2.2.4. Changes in Antioxidant Enzyme Activities

**Figure 4:**
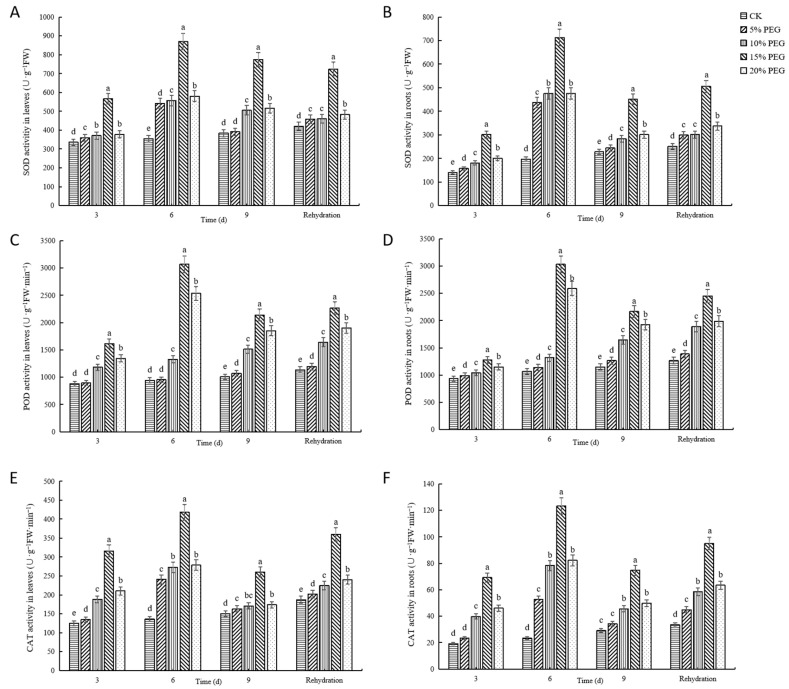
The effects of drought stress induced by different concentrations of polyethylene glycol (PEG) on antioxidant enzyme activities in passion fruit seedlings: (**A**) superoxide dismutase (SOD) activity in leaves. (**B**) SOD activity in roots. (**C**) peroxidase (POD) activity in leaves. (**D**) POD activity in roots. (**E**) catalase (CAT) activity in leaves. (**F**) CAT activity in roots. Different lowercase letters in the same column indicate significant differences among treatments at the 0.05 level.

To evaluate the impact of drought stress on the antioxidant enzyme system, we measured the activities of key antioxidant enzymes, including superoxide dismutase (SOD), peroxidase (POD), and catalase (CAT) (Figure 4), in both the leaves and roots of passion fruit seedlings. SOD activity increased in response to drought stress, reaching a maximum on day 6, and then decreased after rehydration. The highest SOD activity was observed in 15% PEG-6000 treatment at 721.88 U·g^−1^ FW, indicating that passion fruit seedlings responded to drought stress by enhancing their SOD activity.

POD activity showed a differential response to different PEG concentrations. In 10% PEG-6000 treatment, the POD activity gradually increased, while in the 15% PEG-6000 and 20% PEG-6000 treatments, it initially increased sharply and then decreased. The highest POD activity was observed in 15% PEG-6000 treatment on day 6, with 112.75% and 88.36% increases in POD activity in the leaves and roots, respectively, compared with the CK. The results suggest that the effect of drought stress on POD activity is dependent on the intensity and duration of the stress.

Similar to SOD activity, CAT activity showed an increase followed by a decrease in response to drought stress. CAT activity peaked on day 6 in all treatments, with the highest activity in 15% PEG treatment, indicating that CAT plays an important role in protecting passion fruit seedlings from drought.

#### 2.2.5. Changes in Malondialdehyde Content

**Figure 5:**
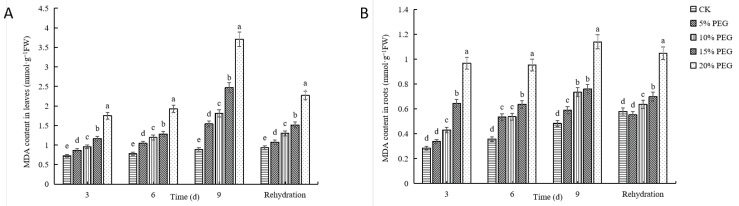
The effects of drought stress induced by different concentrations of polyethylene glycol (PEG) on malondialdehyde (MDA) content in passion fruit seedlings: (**A**) MDA content in leaves. (**B**) MDA content in roots. Different lowercase letters in the same column indicate significant differences among treatments at the 0.05 level.

To investigate the influence of drought stress on cell membrane damage, we quantified the MDA content in both the leaves and roots of passion fruit seedlings (Figure 5). We found that MDA content increased significantly in response to different PEG concentrations, with the highest amount in 20% PEG-6000 treatment, followed by 15% PEG-6000, 10% PEG-6000, and 5% PEG-6000 treatments. The highest MDA level was observed on day 9 of all treatments. Compared with the control, the MDA content in the leaves of 5% PEG-6000,10% PEG-6000, 15% PEG-6000, and 20% PEG-6000 treatments increased by 19.57%, 40.67%, 91.65%, and 187.47%, respectively, while that in the roots increased by 1.83%, 26.47%, 30.96%, and 96.44%, respectively. These results suggest that the degree of cell membrane damage caused by drought stress is related to the intensity and duration of the stress.

## 3. Discussion

Drought is one of the most important factors restricting agricultural production and seriously affecting crop yield. Moreover, plant growth, as well as physiological and biochemical properties, are usually influenced by drought and rehydration-mediated alterations. Therefore, evaluating the stress tolerance mechanism of passion fruit and its ability to recover from drought stress is important for modern agricultural crop production. Drought stress resulted in a reduction in histone kinase activity and cell division, and cell cycle transition was restricted, resulting in a significant reduction in new cell production. When subjected to drought stress, the morphological structure of the plant changes along with its physiological and biochemical properties [27]. When plants lack water during their nutritional phase, water utilization [28] and growth decrease [29], and leaves wilt, resulting in a decrease in fresh and dry weight. In addition, this causes changes in the membrane structure and permeability. Structural changes impair metabolic processes, inhibit photosynthesis, slow down respiration, break down proteins, increase proline (Pro) accumulation, hinder nucleic acid metabolism, and alter hormone metabolism pathways, ultimately leading to a reduction in yield [28].

Fresh and dry weight are important indicators of plant dry matter accumulation, and the magnitude of their values can reflect whether the plant is absorbing water normally. Earlier reduction in plant biomass due to PEG-induced drought was reported in tomato [29] and sesame [30] plants, and it was more pronounced with increasing concentrations of PEG. Under drought stress, *Triticale hexaploid* L. showed a significant reduction in fresh and dry weight compared with the control group [31]. In our study, we observed a similar reduction in fresh and dry biomass of both the above- and belowground parts of passion fruit seedlings under short-term PEG-induced drought stress compared with the control group, which is consistent with previous findings [32]. These results indicate that drought stress impedes plant growth and leads to a decline in biomass. Additionally, we found that the increase in fresh biomass was smaller as the drought degree increased, indicating greater hindrance of water uptake by passion fruit seedlings under higher drought levels. This is consistent with previous results for tomato, sesame, and safflower seeds [33], suggesting that when plants are exposed to drought stress, their capacity to absorb water decreases, leading to a decrease in the quality of fresh and dry matter masses. As the degree of drought increases, the level of damage also increases. Following rehydration, the fresh biomass of both leaves and roots increased rapidly, but the growth rate was lower than that of the control group. There was no significant difference between the control and the low concentration. However, significant differences were observed between the other concentrations and the control, which is consistent with findings for chickpea seedlings under simulated stress of PEG-6000 [34]. These results suggest that seedlings treated with a low concentration of PEG-6000 can recover to normal after rehydration, while those treated with a high concentration of PEG-6000 cannot grow properly after rehydration.

Plants exhibit yellowing and curling of leaves after drought, with different degrees of wilting. Leaves are the main photosynthetic organs in plants, and chloroplasts are the sites of photosynthesis in green plant leaves. Chlorophyll (Chl) is the most important and effective pigment in photosynthesis [35]. Therefore, Chl content reflects plants’ growing conditions and stress levels. In the present study, we found that the Chl content in passion fruit seedlings gradually decreased with increasing PEG-induced stress, indicating that photosynthesis was impaired, and in turn, Chl content decreased in passion fruit. However, the results of this study also reveal that the Chl content of passion fruit gradually increased with increasing duration at the same PEG concentration. Total Chl reduction due to drought stress was reported in chickpeas [36] and Chinese cork oak [37]. After rehydration, the chlorophyll content of passion fruit increased continuously, but the content of each treatment was lower than that of the control. This may be because passion fruit itself has some adaptations that allow it to resist the damage caused by an external drought environment.

In addition, roots perform essential functions. They directly absorb water and nutrients from the soil [38]; therefore, a well-developed root system provides structural support to aboveground parts of the plant and also plays an important role in recovery from injuries [39]. Water deficit in the plant stimulates the longitudinal growth of the main roots, increases the number of lateral roots, and increases the water absorption area, thus increasing the water absorption capacity of the plant [40,41]. However, with intense or long droughts, lignification of the root system increases, root vitality decreases, and the physiological metabolism of the plant is affected, causing injury or even death. This experiment revealed that the root vitality of passion fruit seedlings decreased with the increase in stress level under drought stress (Figure 2); our results are in agreement with the previous study in [42]. However, the root vitality of passion fruit seedlings under drought stress showed an increasing trend with the prolongation of the stress time. This is in contrast to previous results reported by [43] and can be explained by the following reason: the PEG concentration used in this experiment was relatively low and caused less damage to the plant. Therefore, at this low concentration, passion fruit seedlings also had the ability to enhance their root water absorption capacity to enable them to withstand drought stress. After rehydration, the root vitality of each treatment failed to recover to the normal level, which may be due to the short rehydration time, and the water absorption capacity of the root system did not recover in time, resulting in a root vitality below the normal level.

Elevated ROS can cause peroxidation of pigments and lipids on the cell membrane, leading to higher membrane permeability and functional impairment of cell membranes. MDA has been used as an indicator of oxidative damage [44]. Our study found that the level of oxidative damage increased due to drought stress, as determined by higher MDA levels, in passion fruit seedlings. Cucumber and tomato have previously been documented to sustain increased oxidative damage due to drought [6,7,9]. When suffering from drought stress, plants produce too many reactive oxygen species (ROS). Malondialdehyde (MDA) is a product of membrane lipid peroxidation [45] and an important indicator of various stresses. When plants are subjected to these stresses, a large amount of MDA accumulates, further contributing to the destructive effect of biofilm in plants [46]. Drought stress increased the MDA content in the leaves and roots of passion fruit seedlings, which gradually increased with the degree of drought and the extension of drought time and decreased after rehydration. This shows that the higher the PEG concentration, the longer the water shortage time and the greater the drought damage to the seedlings. This finding is consistent with previous research results on other plants [47,48]. The data indicate that, under drought stress, MDA content increases; this could be due to the high peroxidation of plant cell membranes, the aggravation of cell membrane damage, or the deterioration of cell membrane fluidity and permeability, and thus the change in cell structure and function [49]. Moreover, plant damage increases with increasing drought degree and drought time. After rehydration, the MDA content of passion fruit decreased. Furthermore, there was no significant difference between the MDA content of passion fruit root treated with 5% PEG and CK, but the MDA content of passion fruit root treated with 10% PEG and 15% PEG was slightly higher than CK, indicating that the damage degree of passion fruit was relieved after rehydration and could be restored to normal state at low concentration. This result is consistent with previous studies [50].

Exceeding the total oxidative potential of the cell can cause oxidative stress and impair plant growth [51]. To prevent damage from the accumulation of excessive ROS in plants, peroxidase (POD), superoxide dismutase (SOD), and catalase (CAT) together make up the antioxidant defense system of plants, which removes ROS, repairs damaged cells, and maintains cell activity [52,53]. In the later stage, prolonged drought stress destroys the antioxidant enzyme system and reduces antioxidant enzyme activity in the leaf and root systems. A similar phenomenon occurs in strawberry [54] and quinoa [55] plants, with a slight decrease in CAT, POD, and SOD activity also observed in the later stage in passion fruit, possibly due to the increase in drought stress time.

In addition, Pro and soluble protein (SP) accumulate in plants to promote cell osmotic regulation, detoxify ROS, and protect membrane integrity [56]. The increase in Pro content can reduce the permeability of plants and cause damage to the plant [57]. This study found that Pro and SP contents in the leaves and roots of passion fruit seedlings were positively correlated with drought level and time. Furthermore, SP and Pro levels in passion fruit seedlings were decreased after rehydration; with increasing PEG concentration and the prolongation of time, Pro content gradually increased, which is significantly different from the control. Similar findings were reported by Zgalla et al. [58], where PEG-mediated drought stress altered tomato physiological parameters. The contents of SP and Pro in roots of passion fruit seedlings treated with 5% PEG returned to normal values, which had no significant difference from CK. Since these substances play a role in osmotic regulation, reducing cell permeability, maintaining the stability of membrane structure, and improving the ability of seedlings to withstand desiccation, they can increase plants’ resistance to stress.

## 4. Materials and Methods

### 4.1. Experimental Material Preparation and Drought Stress

The experimental material was *Passiflora edulis* “golden” plant cuttings obtained from Guizhou Minjie Ecological Agriculture Investment and Development Co. to be used as experimental material. The seedlings were then transplanted into pots with dimensions of 15 cm × 10 cm and allowed to grow steadily for three days. Seedlings with approximately ten leaves and similar growth patterns were chosen as experimental seedlings for subsequent analysis. The experiments were conducted in the laboratory at the College of Agriculture, Guizhou University, at an incubation temperature of 25 ± 5 °C in the greenhouse. The seedlings were transplanted and watered thoroughly to ensure soil saturation, and the moisture conditions for each treatment were made consistent by using the weighing method [59]. The experimental treatments were carried out when the soil moisture content of each gradient group naturally decreased to the treatment range. The plants were treated with different concentrations of polyethylene glycol (PEG-6000) supplied by Macklin Company (number P815609): 5% PEG-6000, 10% PEG-6000, 15% PEG-6000, and 20% PEG-6000, with distilled water as the control (CK). The duration of stress was 9 days, and samples were collected at 3 d, 6 d, and 9 d, respectively. Plants were then rehydrated, and samples were collected at 3 d. After each sampling, 150 mL of PEG or distilled water of corresponding volume concentration was poured on the roots of the plants.

### 4.2. Experimental Reagents and Equipment

A UV spectrophotometer (UV-5500, Shanghai Yuan Analysis, Shanghai, China) was used to measure the absorbance during the experiment. Some of the reagents used in the experiment were purchased from Shanghai Aladdin Biochemical Technology Co., Ltd. (Shanghai, China). Additionally, some of the chemicals used in the experiment were manufactured by Shanghai Maclin Biochemical Technology Co., Ltd. (Shanghai, China) and purchased for use.

### 4.3. Determination of Morphological and Physiological Parameters

#### 4.3.1. Determination of Growth Indices

The above- and belowground biomass of passion fruit seedlings was determined by cutting the aboveground parts at a distance of 1 cm from the rootstock. Thereafter, aboveground and belowground parts were weighed separately with an electronic balance.

#### 4.3.2. Determination of Chlorophyll Content

To determine the Chl content, the pigments were extracted from 0.05 g of fresh leaves using 10 mL of ice-cold 80% (*v*/*v*) acetone. The mixture was left to mix overnight, and the supernatant was collected through centrifugation at 12,000 rpm for 10 min. The absorbance of the supernatant was measured at 663, 646, and 470 nm. The Chl concentrations were then calculated using equations plotted by Lichtenthaler [60].

#### 4.3.3. Determination of Root Viability

The root vitality was determined using the 2,3,5-triphenyltetrazolium chloride (TTC) method [61]. Firstly, 0.3 g of fresh root tips was placed in a 10 mL centrifuge tube, and 4 mL of 0.4% TTC solution and 4 mL of 0.1 mol·L^−1^ phosphoric acid buffer (pH 7.0) was added, followed by 2 mL of 1 mol·L^−1^ sulfuric acid obtained from Chongqing Chuandong Chemical Industry (Group) Co., Ltd., Chongqing, China. The mixture was ground with ethyl acetate solution and an appropriate amount of quartz sand from Comeo Chemical Reagent Co., Ltd., Chongqing, China. Finally, ethyl acetate was added to the ground solution, and the absorbance at 485 nm was measured. The amount of TTC reduction was then determined based on a standard curve.

#### 4.3.4. Determination of Osmotic Modulating Substances

Soluble protein (SP) in passion fruit leaves and roots was quantified according to the method previously described [62]. For this purpose, 0.2 g of leaf and root was homogenized in 3 mL of 50 mmol·L^−1^ phosphate buffered saline (PBS) (pH 7.8) and centrifuged at 8000× *g* for 10 min. The assay mixture included 0.1 mL of supernatant, 0.9 mL distilled water, and 5 mL of Coomassie brilliant blue. Bovine serum albumin was used as a standard. The absorbance of each sample was measured at a 595 nm wavelength.

The proline (Pro) content was measured using the method described by Subraman-yam [63], and the sample was homogenized in 5 mL sulfosalicylic acid (3%, *w*/*v*) before centrifuging it at 5000× *g* at 25 °C for 20 min. One milliliter of the supernatant was mixed with 1 mL of ninhydrin and acetic acid. The mixture was placed in a 100 °C water bath and allowed to react for 30 min. After that, 4 mL of toluene was added, and the mixture was shaken for 15 s. The final mixture was allowed to stand at room temperature for 10 min, and the absorbance was measured at a 520 nm wavelength.

#### 4.3.5. Antioxidant Enzyme Activity Assay and Determination of Malondialdehyde Content

The activities of protective enzymes such as superoxide dismutase (SOD), peroxidase (POD), catalase (CAT), and malondialdehyde (MDA) were determined by enzyme extraction method, the estimated values of which were measured using crude enzyme extracts. Leaf and root samples (0.5 g) were rapidly homogenized with 8 mL ice-cold PBS (50 mmol·L^−1^, pH 7.8) containing 1% (*w*/*v*) polyvinyl polypyridine in ice. After that, the homogenate was centrifuged at 12,000 × g for 30 min at 4 ℃. The supernatant was put into 1.5 mL centrifuge tubes and stored as a crude extract at −70 °C for the subsequent determination of enzyme activity and MDA content.

SOD activity was determined using a previously described method [64]. The reaction mixture consisted of 1.5 mL of 50 mmol·L^−1^ PBS (pH 7.8), 0.3 mL of 130 mmol·L^−1^ methionine, 0.3 mL of 750 μmol·L^−1^ nitroblue tetrazolium (NBT), 0.3 mL of 100 μmol·L^−1^ EDTA-Na_2_, 0.3 mL of 20 μmol·L^−1^ riboflavin, 0.05 mL of supernatant, and 0.25 mL of distilled water. The reduction of NBT by photoreduction was measured at 560 nm, and SOD activity was determined as the amount of enzyme that inhibited the NBT reduction rate by 50% per unit. POD activity was determined by previously described methods with slight modifications [65]. The reaction system was 0.05 mL of supernatant and 2.95 mL of PBS (100 mmol·L^−1^, pH 6.8) with 1.65 μL of guaiacol and 0.56 μL 30% H_2_O_2_ (Chengdu Jinshan Chemical Reagent Co., Ltd., Chengdu, China). The results were recorded with the increase in absorbance at 470 nm for 4 min and expressed as 0.1 U·mg^−1^ FW.

CAT activity was measured using the ultraviolet absorption method at 240 nm absorbance, as described [65]. The reaction mixture included 2.85 mL of 50 mmol·L^−1^ PBS (pH 7.0), 0.05 mmol·L^−1^ H_2_O_2_, and 0.1 mL of supernatant. The change in absorbance at 240 nm was recorded for 4 min, and the results are expressed as 0.01 U·mg^−1^ FW.

The MDA content was determined using previously described methods [66]. The reaction mixture contained 1 mL supernatant and 2 mL of 0.6% thiobarbituric acid (10% trichloroacetic acid *w*/*v*). After thoroughly mixing the reaction solution, the sample was placed in a boiling water bath for 15 min, cooled rapidly in ice water, and centrifuged at 4000× *g* at 25 °C for 15 min. The absorbance of the supernatant at 532, 600, and 450 nm was recorded.

### 4.4. Statistical Analysis

Microsoft Excel 2010 was used for data processing. The least significant difference test was conducted using the statistics software SPSS, v25.0 (IBM Inc., Chicago, IL, USA) to compare the difference between treatments (*p* < 0.05).

## 5. Conclusions

Treatment with different concentrations of polyethylene glycol (PEG-6000) had substantial effects on the growth and physiology of passion fruit seedlings. The physiological characteristics of passion fruit seedlings changed under drought stress induced by PEG-6000. Similarly, their chlorophyll (Chl) content was inhibited, their antioxidant enzyme activity increased, and the osmoregulatory substance content and root vitality gradually increased. The relative conductivity, malondialdehyde (MDA) content, soluble protein content (SP), and free proline (Pro) content increased with the increase in stress concentration and duration and had a significant difference from CK (*p* < 0.05). The growth rate of fresh biomass and chlorophyll content was considerably reduced, whereas peroxidase (POD), superoxide dismutase (SOD), and catalase (CAT) activities showed an increasing trend followed by a decreasing trend. Different concentrations of PEG have different effects on passion fruit, with 20% PEG treatment having a significant effect on the passion fruit seedlings. Altogether, our study revealed the physiological and biochemical changes in passion fruit under drought stress. In the future, this study will help to explore passion fruit’s drought resistance mechanism. Further molecular and artificial interventions can be used to mitigate the effects of drought on passion fruit.

## Figures and Tables

**Table 1:** The effects of drought stress induced by different concentrations of polyethylene glycol (PEG) on the fresh weight of passion fruit seedlings (CK (control)). Different lowercase letters in the same column indicate significant differences among treatments at 0.05 level.

Treatments		Fresh Weight (g)			
		3 d	6 d	9 d	Rehydration
Root	Control	1.24 ± 0.12 a	1.56 ± 0.20 a	1.75 ± 0.13 a	2.02 ± 0.12 a
	5% PEG-6000	1.20 ± 0.14 a	1.51 ± 0.13 a	1.60 ± 0.11 ab	1.87 ± 0.14 a
	10% PEG-6000	1.04 ± 0.10 ab	1.26 ± 0.13 ab	1.35 ± 0.20 bc	1.44 ± 0.12 b
	15% PEG-6000	0.88 ± 0.09 b	1.02 ± 0.29 b	1.09 ± 0.25 c	1.32 ± 0.10 b
	20% PEG-6000	0.85 ± 0.10 b	0.97 ± 0.13 b	1.02 ± 0.30 c	1.23 ± 0.12 b
Leaf	Control	3.97 ± 0.07 a	4.88 ± 0.28 a	5.66 ± 0.27 a	6.37 ± 0.19 a
	5% PEG-6000	3.91 ± 0.04 a	4.67 ± 0.11 a	5.44 ± 0.17 a	6.08 ± 0.22 a
	10% PEG-6000	3.58 ± 0.05 b	4.27 ± 0.23 b	4.97 ± 0.12 b	5.55 ± 0.21 b
	15% PEG-6000	3.34 ± 0.04 c	3.94 ± 0.22 bc	4.60 ± 0.22 bc	4.97 ± 0.22 c
	20% PEG-6000	3.29 ± 0.04 c	3.76 ± 0.18 c	4.23 ± 0.28 c	4.39 ± 0.29 d

## Data Availability

Not applicable.
